# Pointing to the right side? An ERP study on anaphora resolution in German Sign Language

**DOI:** 10.1371/journal.pone.0204223

**Published:** 2018-09-20

**Authors:** Anne Wienholz, Derya Nuhbalaoglu, Nivedita Mani, Annika Herrmann, Edgar Onea, Markus Steinbach

**Affiliations:** 1 Department of German Philology, University of Goettingen, Göttingen, Lower Saxony, Germany; 2 Georg Elias Müller Institute of Psychology, Psychology of Language Research Group, University of Goettingen, Göttingen, Lower Saxony, Germany; 3 Leibniz ScienceCampus Primate Cognition, German Primate Center, Göttingen, Lower Saxony, Germany; 4 Courant Research Centre “Text Structures”, Junior Research Group “Theoretical Linguistics”, University of Goettingen, Göttingen, Lower Saxony, Germany; University of Akron, UNITED STATES

## Abstract

Sign languages use the horizontal plane to refer to discourse referents introduced at referential locations. However, the question remains whether the assignment of discourse referents follows a particular default pattern as recently proposed such that two new discourse referents are respectively assigned to the right (ipsilateral) and left (contralateral) side of (right handed) signers. The present event-related potential study on German Sign Language investigates the hypothesis that signers assign distinct and contrastive referential locations to discourse referents even in the absence of overt localization. By using a semantic mismatch-design, we constructed sentence sets where the second sentence was either consistent or inconsistent with the used pronoun. Semantic mismatch conditions evoked an N400, whereas a contralateral index sign engendered a Phonological Mismatch Negativity. The current study provides supporting evidence that signers are sensitive to the mismatch and make use of a default pattern to assign distinct and contrastive referential locations to discourse referents.

## Introduction

**Fig 1:**
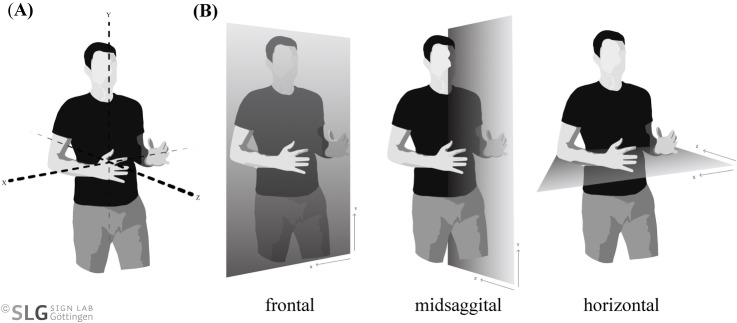
Signing space (A) and its planes (B).

Natural languages come in two modalities, the oral-auditory modality of spoken languages and the visual-gestural modality of sign languages. Although the two modalities clearly differ in production and perception of communicative signals, their underlying linguistic structures seem to be very similar [1–3]. One apparent difference is that sign languages exploit the signing space, which is the space in front of the signer’s upper part of the body and the head (Fig 1A), to realize various phonological, morphosyntactic, semantic, and pragmatic functions.

This space can be subdivided into three different planes [4]: frontal, midsaggital and horizontal (Fig 1B). Crucial for the current study is the horizontal plane, which is used to indicate agreement [5], contrast [6], plurals [7], reciprocals [8], role shift [9,10], temporal reference and topographic relations [11], as well as for linking discourse referents (DRs) [12].

### Discourse referents and referential locations

In spoken languages, a discourse repeatedly refers to certain referents by using anaphoric expressions such as pronouns, e.g., English *he*, *she*, or *it* [13,14]. Similarly, sign language signers use, for instance, spatial pronouns, e.g., pointing with the index finger, to refer back to DRs [3].

Using the signing space to localize DRs varies based on whether referents are physically present or not. For physically present referents, signers can easily identify these by pointing to them and their actual physical location. However, if referents are not physically present, a DR has to be associated with an arbitrary locus on the horizontal plane. This area is called a *referential locus* (henceforth, R-locus; [12,15]). Once a referent is linked to a locus, pronominal signs can be directed toward that locus to refer back to that referent in the subsequent discourse–at least until signers change or overwrite the R-locus associated with this referent [3,12,16]. Various constraints can influence the choice of the locus including semantic or logical relations between referents (e.g., family members may be expressed at the same locus [17]) or pragmatic considerations (i.e., overtly assigning a locus to a referent emphasizes its importance [18]).

Signers can use a variety of overt manual and non-manual strategies, either separately or in combination, to assign referents to an R-locus. One strategy among the manual options that use the hands for referent assignment is pointing with the index finger (index sign) to a R-locus [15,19,20]. In contrast, non-manual strategies include body leans or directing eye-gaze towards a R-locus [19,20]. Moreover, DRs can be implicitly assigned to “default” R-loci, as described in the following section.

### Localizing discourse referents in signing space

Steinbach and Onea [21] discuss a default that appears to govern the assignment of DRs to particular R-loci. First, they note that each DR is linked to a unique and distinct R-locus, which is then used to represent this DR in the discourse representation structure (DRS). Second, they propose the principle of opposition saying that DRs are introduced into maximally contrastive regions in the horizontal space.

Importantly for the purposes of this study, they note that a default pattern, henceforth called right-left default pattern, might constrain the linking of DRs to R-loci. The principle of opposition ensures maximal spatial contrast of the first two DRs. In addition to manual and non-manual strategies signers can use an implicit default strategy that does not include any spatial device. In this case, referents are not explicitly assigned to a particular R-locus. Rather, both the signer and the addressee implicitly associate referents with and refer to this R-locus in subsequent discourse when referring to corresponding DRs. According to the default–at least for right-handed signers–the first referent is typically linked to the right (ipsilateral), and the second to the left (contralateral) side of the signing space.

Support for such a default is reported in several languages, including Catalan, Italian, German and American Sign Language. Thus, in Catalan Sign Language, Barberà [22] observes a tendency for ‘signers (to) use their corresponding ipsilateral part to establish the first location’ (p.103), potentially because this location is closer to the dominant hand (of right-handed signers). In Italian Sign Language, Geraci [23] claims that subjects (first referents since Italian Sign Language is an SOV language) are placed on the side of the dominant hand, such that for right-handed signers, the first referent would be localized on the right side. A similar pattern is reported for a number of languages (German Sign Language (DGS) [9], American Sign Language (ASL) [3,24] and Israeli Sign Language [25]). Studies of other sign languages highlight the use of opposing ipsi- and contralateral sides while remaining silent about positioning first and second DRs to particular sides (British Sign Language [26] and Danish Sign Language [18]).

### Antecedent reactivation in spoken and sign languages

Several studies have investigated antecedent (re-)activation during processing of referential expressions in spoken languages. For instance, in their review Nicol and Swinney [27] show that antecedent reactivation is influenced either by structural (e.g., syntactic relations between antecedent and referential expression) or pragmatic (e.g., salience) information during processing of either explicit (e.g., pronouns) or implicit (e.g., null pronouns) anaphoric expressions. They emphasize that especially structural information cause immediate reactivation of matching antecedents. In case of ambiguous antecedents, all possible referents are simultaneously activated and pragmatic information helps to identify the correct antecedent, although this applies only at a later stage of comprehension (cf. [27] for further references).

For sign languages, Emmorey et al. [28] suggest that antecedent activation appears to require more time relative to spoken languages. They presented signed sentences of ASL containing two possible referents and measured the response times to probe signs presented immediately or with a delay after the critical sign (pronoun or nominal referent) in a second sentence. Response times to probe signs only differed at the later probe sign presentation with faster responses to the pronoun. These results suggest that corresponding referents of the pronoun have only been activated at the delayed probe sign presentation, although the authors suggest that referent activation may occur earlier than measured in the current task [29]. Indeed, subsequent experiments suggested that non-antecedent suppression occurs earlier in sign languages [30]. The authors suggest that this effect may be due to the unambiguous nature of pronouns in ASL, given that pronouns refer to unique spatial locations associated with referents in sign language [28].

Therefore, Emmorey and Falgier [31] investigated the case of *locus doubling* (i.e., associating a single referent with two distinct spatial loci) to examine whether pronouns in ASL activate both their antecedents and the corresponding spatial locations. They presented sentences that associated a referent with two distinct spatial locations continuing with a second sentence containing a pronoun or no anaphoric element and measured response times to probe signs (referent and both locations). Results showed faster response times to referent probes following a pronoun compared to a control, but no difference for both location probes. The findings suggest that ASL pronouns only activate their antecedent but not the spatial location the antecedent is associated with.

Taken together, these studies suggest that pronouns (either overt or phonologically empty) reactivate their antecedent referents [28,32], but not their spatial location [31], thus providing evidence for similarity of antecedent activation in sign and spoken languages. Based on these findings, Emmorey [33] suggests that processing mechanisms involved in resolving and interpreting co-referential relations are the same cross-linguistically and therefore modality-independent. Nevertheless, these studies highlight differences in the timing of antecedent activation and non-antecedent suppression across sign and spoken languages requiring separate investigation.

### The current study

The current study investigates to which extent a default pattern of linking DRs to R-loci in the horizontal plane exists and whether this default pattern influences processing of DRs in DGS. We examine whether signers assign distinct and contrastive R-loci to different DRs and, critically, whether right-handed signers implicitly and automatically assign the first referent to the right (ipsilateral) and the second referent to the left (contralateral) side.

Participants are presented with short discourses, which included introducing two new DRs in the first sentence (e.g., man and woman (By convention, signs are glossed in small caps) (S1 Text) in the subsequent examples–for video stills see S1 Fig). Neither referent is explicitly assigned to an R-locus–thus, the addressee has to rely on default patterns governing the assignment of DRs to R-loci. Afterwards, a second sentence begins with the pointing sign index directed to either the right/ipsilateral (index3a) or left/contralateral (index3b) side. The index functions as a pronoun in this case and, based on whether it is directed to the ipsilateral or contralateral side, anaphorically picks up a DR that has been previously assigned to the respective R-locus. Then a semantically neutral sign (have) and a semantically biased sign (beard), that is only consistent with one of the referents in the first sentence (man), and inconsistent with the other one (woman), follow.

Condition 1:

man woman flirt. index3a have beard.

‘A man flirts with a woman. He has a beard.’

Condition 2:

man woman flirt. index3b have beard.

‘A man flirts with a woman. She has a beard.’

Condition 3:

woman man flirt. index3a have beard.

‘A woman flirts with a man. She has a beard.’

Condition 4:

woman man flirt. index3b have beard.

‘A woman flirts with a man. He has a beard.’

Given reports of the right-left default pattern, we hypothesize that participants implicitly assign the first referent to the ipsilateral side, i.e., man would be assigned to the ipsilateral locus (index3a) in Conditions 1 and 2. By contrast, woman would be assigned to the ipsilateral locus (index3a) in Conditions 3 and 4. Thus the disambiguating noun (beard) would be consistent with the antecedent in Conditions 1 and 4. Based on the default pattern, the ipsilateral locus (index3a) would have been assigned to man in Condition 1 and the contralateral locus (index3b) in Condition 4. In contrast, the disambiguating noun (beard) is inconsistent with the antecedent in Conditions 2 and 3. Here, the default would predict that the ipsilateral locus (index3a) is assigned to woman in Condition 2 and the contralateral locus (index3b) in Condition 3.

Taken together, the prediction that signers automatically assign referents to R-loci based on the default principle implies that they are sensitive to the mismatch between the semantic continuation (beard) and the antecedent (woman) referred to by index in Conditions 2 and 3, relative to Conditions 1 and 4 (man). To test this hypothesis, we record participants’ electroencephalogram (EEG) as they watch videos of signed sentences following the structure outlined above. We anticipate that participants’ sensitivity to semantic mismatch will modulate three event-related potential (ERP) components, the Phonological Mismatch Negativity (PMN), the N400 and the P600.

The earliest component of interest is the PMN, which is thought to reflect phonological processing sensitive to expectations raised by the prior semantic context [34–36]. For instance, Connolly and Phillips [34] report an early negativity (between 150–300 ms) in response to sentence final words phonologically inconsistent with the semantic context thus far. This component typically occurs with a fronto-central distribution [37,38] (but cf. [34]). The N400 is a negative-going component with a broad distribution peaking between 200 and 500 ms following stimulus onset (for a review, see [39,40]). It indexes a complex of processes related to various aspects of meaning processing such as accessing and selecting conceptual representations from semantic memory and integrating them with existing contextual information [41]. Its amplitude is modulated by the ease of semantic processing and increases when meaningful processing is hindered, such as, when a word is incongruent with the preceding semantic context. The P600 is a later positive-going component with a centro-parietal distribution peaking around 600 ms post-stimulus. While it was originally considered to index syntactic processing and reanalysis [42–44], accumulating evidence highlights its sensitivity to semantic anomalies. Münte et al. [45] suggest that the P600 indexes error monitoring and reanalysis. Vissers et al. [46] and van de Meerendonk et al. [47] interpret this component according to a monitoring theory of language perception [48], where comprehenders generate expectancies about upcoming information and monitor incoming information for errors. When linguistic input does not match the expected input, reanalysis is triggered due to mismatching representations. The strength of the conflict reflected in the amplitude varies with the degree of mismatch between expected and encountered events.

All three components have been investigated in sign language processing. For instance, Kutas et al. [49] compared ERPs to sentences ending with a semantic violation for written and spoken English in hearing subjects and for ASL in deaf subjects. For each group, semantically anomalous sentences elicited an N400 effect with slight variations in the onset of the effect. The authors claim that underlying processing of semantic anomalies is similar in reading and listening to English and, crucially, processing of ASL. Similarly, Capek et al. [50] presented deaf participants with signed ASL sentences containing either no violations, a semantic or a syntactic violation. Semantically anomalous signs elicited an N400 relative to baseline condition broadly distributed over posterior regions. Additionally, syntactic violations elicited a left anterior negativity followed by a P600.

Further studies showed similar effects for DGS. Hänel-Faulhaber et al. [51] investigated ERP patterns of signers in reaction to semantically or morphosyntactically anomalous sentences. Morphosyntactic violations evoked an early left anterior negativity followed by a broadly distributed positivity (P600), which can be seen as a typical biphasic pattern (similar to [50]). Semantic violations, in contrast, elicited an N400 effect over fronto-central regions. Hosemann et al. [52] presented participants with sentences ending with a semantically expected or unexpected sign. Unexpected signs evoked an N400 effect in line with Hänel-Faulhaber et al. [51]. However, this effect was found in the transition phase, i.e., the phase between two signs, suggesting that this phase already contains enough information to trigger detection of semantic anomalies even before the onset of the critical sign. Finally, regarding the PMN, while some studies suggest that this component may be restricted to the auditory modality [50,53], Hosemann et al. [52] suggest that their findings may also be compatible with early negativities to phonological mismatches with an expected sign, although they do not explicitly refer to this early negativity as a PMN.

Against this background, we predict that participants will show increased early negative deflections in brain activity to semantically mismatching signs in Conditions 2 and 3, where the antecedent implicitly assigned to the R-locus (per default principle) conflicts with the continuing semantic information. Relative to that, Conditions 1 and 4, where the implicitly assigned antecedent is consistent with the continuing semantic information, will not show negative deflections. We predict this mismatch to trigger reanalysis of the preceding input indexed by a modulation of the P600 component with increased positivity in Conditions 2 and 3 relative to Conditions 1 and 4. Such a pattern would provide strong support for the automatic assignment of referents to R-loci based on the default principle.

## Methods

### Participants

**Fig 2:**
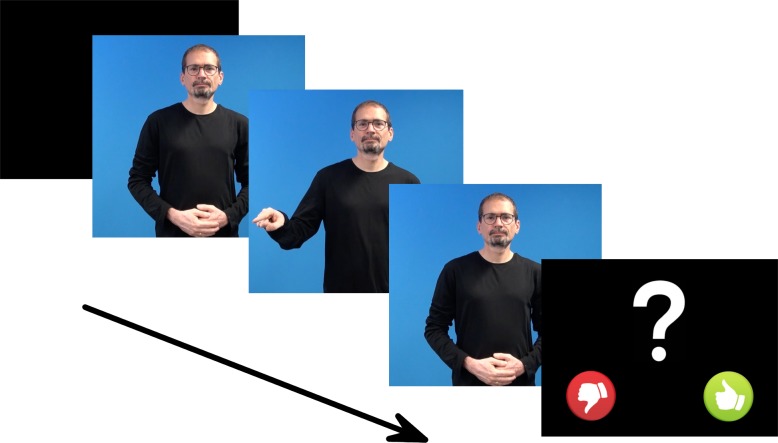
Structure of a single trial.

Twenty-one deaf native signers of DGS from different regions of Germany (12 female, 9 male, age range: 20–51 years, mean: 33 years) participated in the experiment. Eighteen participants were congenitally deaf and 3 deafened later (between 16 and 36 months of age). One participant was excluded from the analysis due to excessive eye movement artifacts. All participants had deaf parents or exposure to DGS before the age of three, were right-handed (as verified by a handedness-test), had normal or corrected-to-normal vision and at least high school education level. Participants were volunteers, paid for their participation and tested in the Experimental Sign Language Lab at the University of Goettingen. All subjects gave written consent before the experiment. Ethics approval was provided by the Ethics Committee of the Institute of Psychology at the University of Goettingen. The model presenting the sign language stimuli pictured in Fig 2 has given written informed consent (as outlined in the PLOS consent form) to publish these case details.

### Material

Forty experimental stimuli sets for each critical condition were developed resulting in 160 experimental stimuli (see S1 Table). Each stimulus set (as presented in 1.4) contained two natural sentences with no causal relation in keeping with the following structure.

The first sentence contained three signs in the neutral SOV word order. Two DRs were introduced without any kind of localization combined with a transitive non-localizing verb. As referents, we used animate entities, i.e., humans or domestic animals [54], to ensure we had combinations where a) we could switch the order of referents without altering semantic and syntactic appropriateness of sentences, b) both referents could act as subject/agent, and c) both referents were equally salient. No compound signs or proper names, requiring either fingerspelling or a fixed name sign, were used because they could potentially increase processing load. Each referent occurred maximally ten times and each referent combination maximally twice. The verbs were transitive and did not localize referents in space. This excluded agreement verbs, which use the horizontal space for marking person and number agreement [55], and some plain verbs, which obligatorily require an additional person agreement marker [56]. Additionally, the verbs chosen allowed switching the order of referents without impacting grammaticality or semantic appropriateness. Each verb was repeated maximally four times and each noun pair-verb combination only occurred once.

The second sentence contained three signs starting with an index followed by two other signs. The index always occurred in subject position but varied in its direction towards an R-locus. The index functioned as a pronoun referring to the referent assigned to either the ipsilateral or contralateral locus, across conditions. The second sign, either an adjective, adverb, noun or possessive pronoun, was semantically neutral. The critical sign in sentence-final position referred back to one of the two referents and was semantically incompatible with the other referent. Assuming that participants automatically apply the right-left default pattern, led to the four conditions described above. Note again that no referent was explicitly assigned to either of these loci in the first sentence, and the second sentence was designed to examine which referent that participants implicitly assigned to a particular R-locus was picked up by the index.

The stimulus material was tested in a questionnaire study with 39 hearing native speakers of German (students at the University of Goettingen) to ensure that the second sentence exclusively refers to one of the previous referents. Here, participants were presented with 92 sentence sets (in written German) that contained a gap at the beginning of the second sentence. Participants were asked to fill the gap with a matching pronoun, to circle the corresponding referent and to rate the certainty of their pronoun choice. 18 participants were excluded from the analysis because they were not native speakers, filling in the questionnaire twice or not completing the task correctly. We chose 40 sentence sets that showed the clearest referent choice and highest certainty ratings as final stimulus sets.

For the actual study, stimuli were interspersed with 80 filler sentences. The material was recorded on video with two male deaf native signers of DGS using a Sony HDR-CX550VE camcorder capturing frontal view. We controlled for the usage of non-manuals to exclude any kind of overt spatial localization such as eye-gaze, body-movements, head-nods or eyebrow raise since not much is known about the influence of non-manuals on anaphora processing in sign languages. However, lexical non-manuals, including mouthing, were allowed because these serve as an important lexical disambiguation device in DGS [57]. There was a natural prosodic break between the two sentences of 108 ms on average (40 ms– 400 ms; no significant differences across conditions) and all verbs were signed in the neutral space in front of the signer.

Videos were cut and processed with Adobe Premiere Pro CS6. The signer remained motionless for 2000 ms at the beginning and 1500 ms at the end of each video. Videos had a size of 1280x720 pixels and a duration of 7000 ms to 9320 ms (mean: 8550 ms). Video duration did not differ significantly across groups (Condition 1: 8533 ms, Condition 2: 8574 ms, Condition 3: 8544 ms, Condition 4: 8548 ms).

Additionally, a set of six practice items was developed with four items similar to the experimental stimuli and two being similar to the filler sentences. The sign combinations were not used during the experiment. They were recorded the same way as sentences in the experiment.

### Pretest and posttest

Additional tests were conducted to ensure that participants knew the crucial signs of the stimuli given possible regional variations of DGS [58,59]. In a pretest, referents from all four conditions used in the first sentence were presented as single signs with their corresponding meaning (German word) as a subtitle. Participants were asked to indicate via button press whether they relate the sign to the presented meaning. In a posttest, participants were asked to write down the meaning of the final signs of the second sentence presented as videos of individual signs in isolation. Since the analysis of ERPs was triggered to this sign, understanding of these signs was checked following the experiment to exclude modulations of the data. Familiarity of each participant with all items and familiarity of each item to participants were evaluated. Participants on average knew 97% of the items (range: 74–100%) in the pretest and on average 97% (range: 84–100%) in the posttest. Items were familiar to participants on average 97% (range: 76–100%) in pretest and on average 95% (range: 71–100%). Thus, no participant and no item were excluded from the analysis based on both tests.

### Procedure

Testing took place in the Experimental Sign Language Lab at the University of Goettingen with the help of a native DGS signer to ensure a DGS language setting. Participants were first informed about the procedure and the EEG technique. All explanations were given in written form and sign language to ensure that all subjects understood the provided information. Then, participants gave their signed consent, filled in a metadata form and participated in a handedness test (www.sign-lang.uni-hamburg.de/dgs-korpus). Participants then took part in the pretest, followed by the experimental session and finally, the posttest.

The experiment took place in a top-lit cabin. Participants were seated on a chair in front of a 24-inch screen at approximately 1 m distance from the screen. Stimuli were presented using the experimental software Presentation, Version 18.1 (Neurobehavioral Systems, Inc.). At the beginning, an introduction video in DGS explained the procedure followed by a practice session to familiarize participants with the trial structure (Fig 2). Participants could repeat the practice session if required. This was followed by the actual experiment. Each trial began with a blank screen for 500 ms followed by a video, displayed in the center of the screen, showing one sentence set in the speed of natural signing. At the end of each video, a question mark appeared indicating that participants should judge whether the sentence was *good* or *bad* by relying on their first immediate reaction and by pressing either a green ‘thumbs up’ button or a red ‘thumbs down’ button on a Microsoft Xbox 360 Wired Controller. There was no time limit for their response. Participants were instructed to avoid body movement and, if possible, to blink after the video was presented. Between blocks, participants could take breaks and could decide themselves when to continue with the experiment.

Experimental sentence sets were combined into four different lists, pseudo-randomized in 10 blocks of 24 sentence sets each. Each block contained eight filler sentences and 16 experimental stimuli with four sentences from each condition, ensuring that sentences within a block were all from different sentence sets. The order within a block was randomized on-line.

### EEG-recording

We recorded EEG data using 64 active Ag/AgCl electrodes placed according to the international 10–20 system and data were amplified using an ActiveTwo AD-Box (BioSemi B.V., Amsterdam, Netherlands) with a sampling rate of 2048 Hz. Data were re-referenced off-line to linked mastoids. Impedances were kept below 20 kΩ. For each participant, the electrooculogram was monitored using three external electrodes, two of which recorded the horizontal and one recorded the vertical electrooculogram in combination with electrode FP2 on the cap. EEG data were analyzed using the ERPLAB toolbox plugin [60] for MATLAB (The MathWorks, Inc.). The EEG signal was filtered using a 0.1–30 Hz band-pass filter and downsampled to 250 Hz. We calculated ERP averages for single subjects for each condition and electrode at different trigger points. Hosemann et al. [52] revealed that processing of signs is triggered even before the sign’s onset is presented (similar to co-articulation information in spoken language). Therefore, we defined three different triggers following Hosemann et al. [52].

1. Sign offset: Last frame of the final hold of the sign preceding the critical sign before the hand gets relaxed again and before the movement of the transition phase starts. This trigger was used for baseline correction.
2. Target handshape: First frame in which the target handshape was identifiable, regardless of target orientation or location. On average, the target handshape trigger position occurred 146 ms after the sign offset2 trigger position.
3. Sign onset: First frame of the critical sign where the target hand configuration reached its target location and the hands were about to start the path movement. On average, the sign onset trigger position occurred 202 ms after the target handshape trigger position. The duration of the critical sign, defined as duration from sign onset to sign offset, was on average 384 ms.

These trigger positions were manually identified by two independent coders for each video using the ELAN software [61]. ERPs were timelocked separately to target handshape and sign onset triggers and corrected for baseline activity 200 ms prior to sign offset triggers of the final disambiguating sign in the second sentence. Trials with blinks or other artifacts were rejected before averaging with an artifact rejection threshold of 100 μV. Preliminary analyses run on 100 ms epochs across trials revealed significant differences between conditions following the target handshape trigger between 200–300 ms and 500–600 ms in keeping with the early PMN and N400 time windows. However, no time window associated with the P600 revealed significant differences. We, therefore, focused on the two significant time windows for further analyses. Additional analyses on time windows following the sign onset trigger revealed no significant differences. All EEG and behavioral datasets underlying the analysis in the current study are available using the following DOI: 10.17605/OSF.IO/NBMRJ deposited in the Open Science Framework.

## Results

### Behavioral data

Nineteen participants were included in the judgement data analysis (one participant was excluded as described in the methods section and one participant was excluded due to technical problems). Mean judgement ratings obtained from participants for each condition are reported in Table 1. There were no differences in participants’ judgements of sentence acceptability across conditions (*p* > .1).

**Table 1:** Mean judgement ratings and standard deviations for each condition.

Condition	Mean	Standard deviation
1	1.45	0.31
2	1.45	0.32
3	1.47	0.31
4	1.39	0.31

For means, 1 indicates a rating as ‘good’ and 2 indicates a rating as ‘bad’.

### ERP data

For evaluation, we analyzed the identified time windows, i.e., 200–300 ms and 500–600 ms, in two different ways. In the following, only significant and near-significant results are reported.

First, data from Condition 1 with 4 and Conditions 2 with 3 were combined to examine whether participants automatically assigned the first referent to the ipsilateral locus. This would render the referent congruent with the disambiguating sign in Conditions 1 and 4, and incongruent in Conditions 2 and 3, thus focusing on congruence or incongruence between disambiguating sign and referent assigned to the locus regardless of what this locus (index3a / index3b) is.

**Fig 3:**
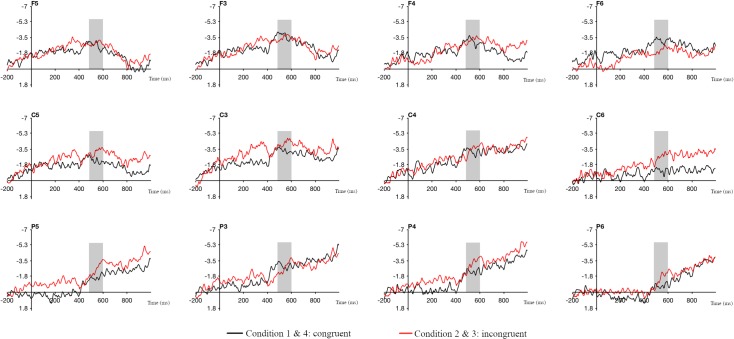
Grand average ERPs for congruent (black) and incongruent (red) conditions. Data are timelocked to the target handshape trigger. C3 and C5 are central right and left lateral electrodes on the left hemisphere. C4 and C6 are central left and right lateral electrodes on the right hemisphere. Negativity is plotted upwards.

Data from lateral electrodes (F1-8, C/T1-8, P1-8,) were split by region and hemisphere, and analyzed with a 4x3x2x2 ANOVA with the factors laterality (far-left, left, right, far-right), region (frontal, central, parietal), hemisphere (left, right) and match (congruent, incongruent). The Greenhouse-Geisser correction [62] was applied as required. Visual data inspection suggested a sustained negativity across the two conditions, with increased negative activity to incongruent relative to congruent conditions (Fig 3). However, analysis across these conditions yielded no significant differences in the early time window (200–300 ms).

Analysis of the later time window, from 500–600 ms, revealed a significant four-way interaction [F(6,114) = 3.041; *p* = .009], an interaction between hemisphere*laterality*match [F(3, 57) = 3.099; *p* = .034] and a near-significant interaction between region*match [F(2,38) = 3.554; *p* = .055]. Pivoting on laterality revealed a significant three-way interaction between region*hemisphere*match in the data across left [F(2,38) = 5.461; *p* = .008] and right electrode sites [F(2,38) = 3.490; *p* = .041] and a near-significant interaction between region*match across far right electrodes [F(2,38) = 2.974; *p* = .063].

Pivoting on hemisphere, analysis of left hemisphere left electrode sites with the factors region and match revealed a near-significant interaction [F(2,38) = 3.462; *p* = .056], which we broke down to reveal a near-significant difference between congruent [M = -2.798] and incongruent [M = -4.782] conditions across central electrode sites [t(19) = 1.998; *p* = .06]. No significant differences between conditions were found for right hemisphere left electrode sites.

Analysis of right hemisphere right electrode sites with the factors region and match revealed a significant interaction [F(2,38) = 5.992; *p* = .005], which revealed a significant difference between congruent [M = -0.945] and incongruent [M = -2.979] conditions across the central region [t(19) = 2.364; *p* = .029].

A second analysis split conditions depending on whether the locus picked up by the index was the ipsilateral or contralateral locus, i.e., separately compared Conditions 1 to 4, since participants may expect the first referent to be referred to in the second sentence relative to the second referent. This would render Condition 1 more appropriate than Condition 4. Data from lateral electrodes (F1-8, C/T1-8, P1-8,) were split by region and hemisphere and analyzed with a 4x3x2x2x2 ANOVA with the factors laterality (far-left, left, right, far-right), region (frontal, central, parietal), hemisphere (left, right), direction (ipsilateral, contralateral) and match (congruent, incongruent).

**Fig 4:**
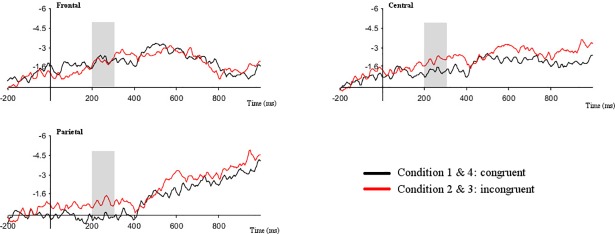
Grand average ERPs congruent (black) and incongruent (red) conditions. Data are timelocked to the target handshape trigger for frontal, central and parietal regions across left and right hemisphere. Negativity is plotted upwards.

Across the early time window, from 200–300 ms, analysis revealed a four-way interaction of hemisphere*region*laterality*match [F(6,114) = 2.797; *p* = .014], a four-way interaction of hemisphere*region*direction*match [F(2,38) = 3.956; *p* = .028] and a four-way interaction of hemisphere*laterality*direction*match [F(3,57) = 3.582; *p* = .019]. It also revealed three-way interactions between hemisphere*region*direction [F(2,38) = 3.468; *p* = .041] and hemisphere*laterality*direction [F(3,57) = 3.943; *p* = .033] as well as interactions between hemisphere*direction [F(1,19) = 5.325; *p* = .032] and region*match [F(2,38) = 6.198; *p* = .014]. Pivoting on region (Fig 4), paired samples t-tests comparing congruent [M = -0.579] and incongruent [M = -1.120] conditions revealed a near-significant difference across central regions [t(19) = 1.891; *p* = .074] and a significant difference between congruent [M = 0.127] and incongruent [M = -0.618] conditions across parietal electrodes [t(19) = 2.322; *p* = .032]. This mirrors the findings in the analyses reported above with overall increased negativity to incongruent relative to congruent trials across parietal and central regions, also in the early time window.

**Fig 5:**
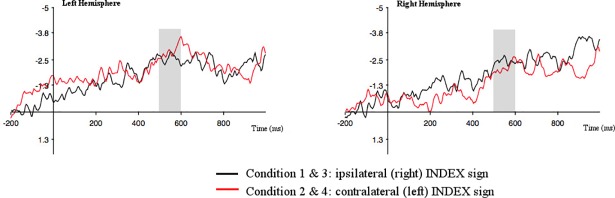
**Grand average ERPs for conditions containing an ipsilateral/right (black) and contralateral/left (red) index**. Data are timelocked to the target handshape trigger for both left and right hemisphere. Negativity is plotted upwards.

Breaking down the interaction between hemisphere and direction (Fig 5), we found increased negative activity in the left relative to the right hemisphere when the index picked up the contralateral locus [t(19) = -4.517; *p* = .000]. This suggests increased processing costs in the left hemisphere when the contralateral direction is processed. In the time window from 500–600 ms, a similar effect was found when the data were grouped by direction regardless of match with increased negative activity across left hemisphere sites relative to right hemisphere sites when the index pointed to the contralateral locus, [t(19) = -2.362; *p* = .029]. No other comparisons yielded significance.

## Discussion

This study investigated to which extent signers employ the right-left default pattern governing the assignment of DRs to R-loci in the horizontal plane in DGS. We examined this by presenting participants with sentences assigning referents implicitly to an R-locus, and a pronoun picking up one referent either consistent or inconsistent with a sentence-final semantically disambiguating sign. Increased negative deflections in the N400 time window indicated that participants indeed applied the proposed pattern of referent assignment, thus confirming our hypotheses. First of all, the influence of congruence of the disambiguating term and antecedent *implicitly* assigned to a locus provides evidence that signers automatically assign distinct and contrastive R-loci to different DRs, since the antecedent referred to was never *explicitly* assigned to this locus. Second, our results suggest that antecedents are not arbitrarily assigned, but rather, that there appears to be a specific default governing the assignment to distinct R-loci. In particular, the results can only be explained by suggesting that right-handed signers indeed apply the right-left default pattern implemented in a modified version of Discourse Representation Theory as proposed by Steinbach and Onea [21]. Thus, the study reveals that, even in the absence of an overt localization strategy, signers construct a spatial structure of the present discourse to guide their understanding of referential expressions. They automatically make use of opposing R-loci in the horizontal plane towards, in this case, anaphoric disambiguation. Therefore, R-loci can be analyzed as a modality-specific grammatical device that is used to structure the set of DRs in the DRS and to restrict the set of possible DRs that a pronominal expression is referring to. Hence, the results provide evidence that DGS exploits the specific properties of the signing space to facilitate reference tracking in signed discourse.

This allows for an interesting parallel between spoken languages like German and DGS, since grammatical gender fulfills a similar function in the processing of many spoken languages. In German, for instance, nouns take one of three genders, i.e., masculine, feminine and neuter. Pronouns agree in grammatical gender with antecedents they refer to (e.g., *der/er* for masculine nouns). In a discourse containing a masculine and a feminine noun, a masculine pronoun unambiguously refers back to the former and speeds up activation of the correct antecedent even before further information is provided (for a review see [63]). This is similar to the role of R-loci in sign language processing since referring to an R-locus retrieves the antecedent assigned to it, thereby facilitating further processing of the discourse. Thus, sign and (some) spoken languages appear to have both developed similar but modality-specific strategies to fulfill the same discourse function.

Additional analyses examined differences between trials within conditions to determine whether participants expect the first referent to be continued with in subsequent discourse. This revealed three interesting findings. First, we found no modulation of ERPs by direction alone within either congruent or incongruent trials. While this might be taken as evidence that participants have no preference for the first DR, we suggest caution in reaching this interpretation until this finding has been replicated in sentences containing referents explicitly assigned to particular loci–it may be that implicitly assigned R-loci are too weak to support such pragmatic constraints on discourse interpretation. Second, we found a modulation of ERPs by congruence of disambiguating term and antecedent implicitly assigned to the R-locus in the early time window. Here, semantic/phonological expectations are violated, thus the modulation reflects participants’ sensitivity. Third, we found a lateralization effect with increased negative deflections in the left hemisphere for the contralateral index, i.e., the left side of the signer (right visual field of participants). We interpret this finding in keeping with reported intrinsic attentional biases of the left hemisphere to the contralateral visual field, with earlier latencies and enhanced amplitude contralaterally for early ERP components (e.g., N2 and P2; [64,65]), rather than effects of violation of phonological expectation, as in the case of the PMN. Nevertheless, this early time window is modulated by congruence of disambiguating sign and antecedent picked up by the R-locus (regardless of the direction of this locus). This suggests different contributors to the modulation of ERPs in this time window, one sensitive to visual field attentional biases and the other sensitive to violations of semantic/phonological expectations. The latter modulation resembles the PMN effect which has, to date, not been reported for sign language processing. Further research is needed to examine the different processes contributing to differences in this early time window and to which extent the PMN is shared across modalities.

Similar to other ASL studies (e.g., [53]) the current N400 component seems to peak later than usual (cf. [40]). Capek et al. [50] argue that the onset of this effect resembles findings observed for written stimuli. The N400 component is usually observed between 300 and 500 ms [40]. However, the N400 effect in the current study occurred in a different time window, later than usual (500–600 ms). A closer look at previous studies on ASL reveals that they show a similarly delayed N400. Gutiérrez et al. [53] observe an N400 between 450–600 ms and Capek et al. [50] similarly report effects of semantic processing in a broad time window from 300 to 875 ms. The authors state that the onset of this effect resembles findings observed for written stimuli. Indeed, effects for auditory conveyed information seem to occur earlier than similar effects evoked by visual information irrespective of whether they examine processing of written or signed stimuli [40,49,66]. Thus, a delayed onset of the N400 might be typical for sign languages due to their modality.

Another potential reason for the delayed N400 may be related to the delay between the onset of our analysis window and the uniqueness point of a sign, i.e., the point when this sign can be recognized. The analyses reported in 3.2 are timelocked to the target handshape trigger, i.e., the first frame in which the target handshape was identifiable. This occurred on average 200 ms before sign onset, i.e., first frame in which the target hand configuration reached the target location and the hands started their path movement. Variability regarding when the sign is recognizable and when processing is advanced enough to trigger a match or a mismatch response, might cause the delay. This might suggest that our current analysis is timelocked to an earlier trigger relative to when detection of a mismatch is possible. However, there are no standardized regulations to determine trigger positions in EEG experiments investigating sign languages. Indeed, Hosemann et al. [52] provided evidence for the important role of the transition phase between two signs for EEG studies. Transition phases are longer and more pronounced in sign languages since they use the signing space to convey information, which results in longer transitions from one sign to the next due to their spatial distribution and relatively large, slow and heavy articulators [1]. Additionally, transitions between signs have different lengths and trajectories that depend on the context and surrounding signs. Therefore, information is available at different time points for each transition and it is hard to determine a universally identifiable trigger position, leading to issues with regard to time locking electrical activity to particular time points in the signal.

An additional point to be considered is the language status of the participants. While all participants were native signers, none of them were monolinguals in the strict sense. Participants are surrounded by written German and thus acquire German as a second language early in life. The bilingual background may influence the timing of expected effects. A number of studies suggest that the N400 effect in bilinguals, while showing a similar distribution, can vary in both amplitude and latency [67–69]. Indeed, in a cross-modal priming study on DGS, Hosemann [70] found that native signers automatically activate the German counterpart of a DGS sign. Such unconscious cross-language cross-modal activation may modulate both behavioral data–leading to slower reaction times–and ERP data–leading to changes with regard to amplitude and latency of particular components [71,72].

Moreover, it is worth discussing the absence of any modulation of the P600 component. As outlined in 1.4, one might expect a similar modulation of the P600 given the semantic anomaly between disambiguating sign and referent. Alternatively, one might expect syntactic reanalysis regarding referent assignment triggered by this disambiguating sign. We suggest two reasons for the absence of such a modulation. First, it is possible that processes of error monitoring abort at some point, due to increased semantic implausibility. This possibility is refuted, however, by studies suggesting that even implausible sentences trigger error monitoring (e.g., [73]). Second, the analysis of the behavioural data showed that sentences were equally acceptable with no significant differences in judgement ratings for any condition. This would suggest that sentences are, from a syntactic perspective, at least, well-formed and would, therefore, not trigger a P600 effect. Indeed, our finding of a modulation of semantic and not syntactic ERP indices supports this suggestion. This would reflect the strength of the default assignment, that possibly can be overwritten easily once the semantic anomaly is detected, mirrored in ERP components modulation.

Finally, it might be surprising that in the behavioural data all conditions were rated similarly even if conditions 2 and 3 were expected to receive lower ratings due to their semantic mismatch compared to conditions 1 and 4. One reason for the similar ratings may be the high variability and the lack of a standardized form of DGS. Thus, participants may have a higher tolerance to the provided language input. Moreover, the language status of participants as possible bilinguals as mentioned above and thus the influence of spoken German might affect their ratings [74]. Lastly, the lack of a difference in the ratings may be due to a pragmatic effect related to anaphora resolution. The behavioural task follows stimulus presentation and thus a (late) context-driven re-interpretation could apply to achieve a successful interpretation of the sentence set. Additional pragmatic factors such as accessibility may apply here as well which requires more evidence from future research.

## Conclusion

By investigating the modality-specific way of introducing DRs and establishing anaphoric relations in DGS, this experimental study improves our understanding of discourse structures in sign languages, it provides new insights into the impact of modality on linguistic structure in particular and on the structure of the human language faculty in general. The present study demonstrated that a) right-handed deaf native signers of DGS assign distinct and contrastive R-loci to different DRs and b) that a specific default pattern governs the R-loci assignment of antecedents. In particular, right-handed signers implicitly assign the first referent to the ipsilateral and the second referent to the contralateral side of the signing space. This suggests that signers employ a specific default strategy for anaphora resolution (space) similar to spoken language users (gender). Such defaults can, however, be overwritten by other factors influencing discourse structuring, such as overt localization, information structural issues, and well-formedness as indicated by participants’ judgements. It remains an open question whether the observed default pattern holds for left-handed signers as well. A difference in the observed pattern would suggest that the location of both sides and terms would be defined in relation to the dominant hand of the signer in line with Geraci [75]. Moreover, the pattern may depend on the handedness of the signer presented in the stimulus material. Ongoing research in our sign language lab is currently assessing this and other factors influencing the processing of DRs in sign language research.

## Supporting information

S1 Text Notational conventions for sign glosses. (PDF)

S1 Fig Scene stills of videos for each condition. (PDF)

S1 Table Full list of stimulus material. (PDF)

## References

[1] Meier RP. Why different, why the same? Explaining effects and non-effects of modality upon linguistic structure in sign and speech. In: Meier RP, Cormier K, Quinto-Pozos D, editors. Modality and Structure in Signed and Spoken Languages. Cambrigde, UK: Cambridge University Press; 2002 pp. 1–25.

[2] Meier RP. Language and Modality In: Pfau R, Steinbach M, Woll B, editors. Sign Language: An International Handbook Berlin, Boston: De Gruyter Mouton; 2012 pp. 574–601.

[3] Sandler W, Lillo-Martin D. Sign Language and Linguistic Universals Cambrigde, UK: Cambridge University Press; 2006.

[4] Brentari D. A Prosodic Model of Sign Language Phonology Cambridge, MA: MIT Press; 1998.

[5] Padden C. The relation between space and grammar in ASL verb morphology In: Lucas C, editor. Sign language research: Theoretical issues. Washington, D.C: Gallaudet University Press; 1990 pp. 118–132.

[6] van der Kooij E, Crasborn O, Emmerik W. Explaining prosodic body leans in Sign Language of the Netherlands: Pragmatics required. J Pragmat. 2006;38: 1598–1614. 10.1016/j.pragma.2005.07.006

[7] Pfau R, Steinbach M. Pluralization in sign and in speech: A cross-modal typological study. Linguist Typology. 2006;10: 135–182. 10.1515/LINGTY.2006.006,

[8] Pfau R, Steinbach M. Optimal reciprocals in German Sign Language. Sign Lang Linguist. 2003;6: 3–42. 10.1075/sll.6.1.03pfa

[9] Herrmann A, Steinbach M. Wenn ‘ich’nicht ich ist: Zitieren in Gebärdensprachen In: Brendel E, Meibauer J, Steinbach M, editors. Zitat und Bedeutung. Hamburg: Buske; 2007 pp. 153–179.

[10] Lillo-Martin D. Utterance reports and constructed action in sign and spoken languages In: Pfau R, Steinbach M, Woll B, editors. Sign Language: An International Handbook. Berlin, Boston: De Gruyter Mouton; 2012 pp. 365–387.

[11] Emmorey K. Language, Cognition, and the Brain: Insights From Sign Language Research Mahwah, NJ: Lawrence Erlbaum and Associates; 2002.

[12] Lillo-Martin D, Klima ES. Pointing Out Differences: ASL Pronouns in Syntactic Theory In: Fischer SD, Siple P, editors. Theoretical Issues in Sign Language Research, Volume 1: Linguistics. Chicago, IL: University of Chicago Press; 1990.

[13] Ariel M. Accessing noun-phrase antecedents London, UK: Routledge; 1990.

[14] Ariel M. Accessibility theory: An overview In: Sanders TJ, Schilperoord J, Spooren W, editors. Text representation: Linguistic and psycholinguistic aspects. Amsterdam, Philadelphia: John Benjamins Publishing Company; 2001 pp. 29–87.

[15] Liddell SK. Four functions of a locus: Re-examining the structure of space in ASL In: Lucas C, editor. Sign language research: Theoretical issues. Washington, D.C: Gallaudet University Press; 1990 pp. 176–198.

[16] Poizner H, Klima ES, Bellugi U. What the Hands Reveal about the Brain Cambridge, MA: MIT Press; 1987.

[17] Klima ES, Bellugi U. The Signs of Language Cambridge, MA: Harvard University Press; 1979.

[18] Engberg-Pedersen E. Space in Danish Sign Language: The Semantics and Morphosyntax of the Use of Space in a Visual Language Hamburg: Signum Verlag; 1993.

[19] Lillo-Martin D. Two kinds of null arguments in American Sign Language. Nat Lang Linguist Theory. 1986;4: 415–444. 10.1007/bf00134469

[20] Winston EA. Spatial mapping in ASL discourse In: Jones DM, editor. Assessing Our Work: Assessing Our Worth. Little Rock, AR: CIT Conference Proceedings; 1996 pp. 1–28.

[21] Steinbach M, Onea E. A DRT Analysis of Discourse Referents and Anaphora Resolution in Sign Language. J Semant. 2016;33: 409–448. 10.1093/jos/ffv002

[22] Barberà G. The Meaning of Space in Sign Language Reference, Specificity and Structure in Catalan Sign Language Discourse. Berlin, Boston: De Gruyter Mouton; 2012.

[23] Geraci C. Spatial syntax in your hands. Linguistic Seminar, CNRS Institut Jean-Nicod; 2013 Jun 19; Paris.

[24] Schlenker P. Anaphora: Insights from Sign Language In: Anderson SR, Moeschler J, Reboul F, editors. The Language-Cognition Interface: Actes du 10e Congrès International des Linguistes. Geneva, Switzerland: Librairie Droz; 2013.

[25] Meir I, Sandler W. A language in space: The story of Israeli Sign Language New York, NY: Lawrence Erlbaum Associates; 2008.

[26] Sutton-Spence R, Woll B. The linguistics of British Sign Language: An Introduction Cambrigde, UK: Cambridge University Press; 1999.

[27] Nicol J, Swinney D. The role of structure in coreference assignment during sentence comprehension. J Psycholinguist Res. 1989;18: 5–19. 10.1007/bf01069043 2647962

[28] Emmorey K, Norman F, O’Grady L. The activation of spatial antecedents from overt pronouns in American Sign Language. Lang Cogn Process. 1991;6: 207–228. 10.1080/01690969108406943

[29] Emmorey K, Corina D. Lexical Recognition in Sign Language: Effects of Phonetic Structure and Morphology. Percept Mot Skills. 1990;71: 1227–1252. 10.2466/pms.1990.71.3f.1227 2087376

[30] Emmorey K. Non-antecedent suppression in American Sign Language. Lang Cogn Process. 1997;12: 103–120. 10.1080/016909697386925

[31] Emmorey K, Falgier B. Conceptual locations and pronominal reference in American Sign Language. J Psycholinguist Res. 2004;33: 321–331. 10.1023/b:jopr.0000035104.83502.0b 15360124

[32] Emmorey K, Lillo-Martin D. Processing spatial anaphora: Referent reactivation with overt and null pronouns in American Sign Language. Lang Cogn Process. 1995;10: 631–653. 10.1080/01690969508407116

[33] Emmorey K. The psycholinguistics of signed and spoken languages: How biology affects processing In: Gaskell G, editor. The Oxford Handbook of Psycholinguistics. Oxford: Oxford University Press; 2007 pp. 703–721.

[34] Connolly JF, Phillips NA. Event-related potential components reflect phonological and semantic processing of the terminal word of spoken sentences. J Cogn Neurosci. 1994;6: 256–266. 10.1162/jocn.1994.6.3.256 23964975

[35] D’Arcy RC, Connolly JF, Crocker SF. Latency shifts in the N2b component track phonological deviations in spoken words. Clin Neurophysiol. 2000;111: 40–44. 10.1016/s1388-2457(99)00210-2 10656509

[36] Newman RL, Connolly JF, Service E, Mcivor K. Influence of phonological expectations during a phoneme deletion task: Evidence from event-related brain potentials. Psychophysiology. 2003;40: 640–647. 10.1111/1469-8986.00065 14570171

[37] Connolly JF, Phillips NA, Stewart SH, Brake WG. Event-related potential sensitivity to acoustic and semantic properties of terminal words in sentences. Brain Lang. 1992;43: 1–18. 10.1016/0093-934x(92)90018-a 1643505

[38] Connolly JF, Stewart SH, Phillips NA. The effects of processing requirements on neurophysiological responses to spoken sentences. Brain Lang. 1990;39: 302–318. 10.1016/0093-934X(90)90016-a 2224497

[39] Kutas M, Federmeier KD. Electrophysiology reveals semantic memory use in language comprehension. Trends Cogn Sci. 2000;4: 463–470. 10.1016/s1364-6613(00)01560-6 11115760

[40] Kutas M, Federmeier KD. Thirty years and counting: finding meaning in the N400 component of the event-related brain potential (ERP). Annu Rev Psychol. 2011;62: 621–647. 10.1146/annurev.psych.093008.131123 20809790 PMC4052444

[41] Lau EF, Phillips C, Poeppel D. A cortical network for semantics: (de)constructing the N400. Nat Rev Neurosci. 2008;9: 920–933. 10.1038/nrn2532 19020511

[42] Hagoort P, Brown C, Groothusen J. The syntactic positive shift (SPS) as an ERP measure of syntactic processing. Lang Cogn Process. 1993;8: 439–483. 10.1080/01690969308407585

[43] Kaan E, Harris A, Gibson E, Holcomb P. The P600 as an index of syntactic integration difficulty. Lang Cogn Process. 2000;15: 159–201. 10.1080/016909600386084

[44] Münte TF, Matzke M, Johannes S. Brain activity associated with syntactic incongruencies in words and pseudo-words. J Cogn Neurosci. 1997;9: 318–329. 10.1162/jocn.1997.9.3.318 23965010

[45] Münte TF, Heinze H-J, Matzke M, Wieringa BM, Johannes S. Brain potentials and syntactic violations revisited: No evidence for specificity of the syntactic positive shift. Neuropsychologia. 1998;36: 217–226. 10.1016/s0028-3932(97)00119-x 9622187

[46] Vissers CTW, Chwilla DJ, Kolk HH. Monitoring in language perception: The effect of misspellings of words in highly constrained sentences. Brain Res. 2006;1106: 150–163. 10.1016/j.brainres.2006.05.012 16843443

[47] van de Meerendonk N, Indefrey P, Chwilla DJ, Kolk HH. Monitoring in language perception: Electrophysiological and hemodynamic responses to spelling violations. Neuroimage. 2011;54: 2350–2363. 10.1016/j.neuroimage.2010.10.022 20955801

[48] van de Meerendonk N, Kolk HH, Chwilla DJ, Vissers CTW. Monitoring in language perception. Lang Linguist Compass. 2009;3: 1211–1224. 10.1111/j.1749-818X.2009.00163.x

[49] Kutas M, Neville HJ, Holcomb PJ. A preliminary comparison of the N400 response to semantic anomalies during reading, listening and signing. Ellingson RJ, Murray NMF, Halliday AM, editors. Electroencephalogr Clin Neurophysiol Suppl. 1987; 325–330. 3477442

[50] Capek CM, Grossi G, Newman AJ, McBurney SL, Corina D, Röder B, et al Brain systems mediating semantic and syntactic processing in deaf native signers: Biological invariance and modality specificity. Proc Natl Acad Sci. 2009;106: 8784–8789. 10.1073/pnas.0809609106 19433795 PMC2689005

[51] Hänel-Faulhaber B, Skotara N, Kügow M, Salden U, Bottari D, Röder B. ERP correlates of German Sign Language processing in deaf native signers. BMC Neurosci. 2014;15: 62 10.1186/1471-2202-15-62 24884527 PMC4018965

[52] Hosemann J, Herrmann A, Steinbach M, Bornkessel-Schlesewsky I, Schlesewsky M. Lexical prediction via forward models: N400 evidence from German Sign Language. Neuropsychologia. 2013;51: 2224–2237. 10.1016/j.neuropsychologia.2013.07.013 23896445

[53] Gutiérrez E, Williams D, Grosvald M, Corina D. Lexical access in American Sign Language: An ERP investigation of effects of semantics and phonology. Brain Res. 2012;1468: 63–83. 10.1016/j.brainres.2012.04.029 22763237

[54] Murmann C. The agreement auxiliary PAM in German Sign Language—An empirical investigation. Master’s Thesis, Heinrich Heine Universität. 2012.

[55] Mathur G, Rathmann C. Verb agreement In: Pfau R, Steinbach M, Woll B, editors. Sign Language: An International Handbook. Berlin, Boston: De Gruyter Mouton; 2012.

[56] Rathmann C. The optionality of agreement phrase: Evidence from German Sign Language (DGS) In: Griffin WE, editor. The role of agreement in natural language: Proceedings of the Fifth Annual Texas Linguistics Society Conference. Austin, TX: Texas Linguistic Forum; 2003 pp. 181–192.

[57] Ebbinghaus H, Heßmann J. Sign language as multidimensional communication: Why manual signs, mouthings, and mouth gestures are three different things In: Boyes Braem P, Sutton-Spence R, editors. The hands are the head of the mouth. Hamburg: Signum Verlag; 2001 pp. 133–151.

[58] Eichmann H, Rosenstock R. Regional variation in German Sign Language: The role of schools (re-) visited. Sign Lang Stud. 2014;14: 175–202. 10.1353/sls.2014.0001

[59] Langer G. A colorful first glance at data on regional variation extracted from the dgs-corpus: with a focus on procedures. 5th Workshop on the Representation and Processing of Sign Languages: Interactions between Corpus and Lexicon. Istanbul; 2012. pp. 101–108. Available: https://www.sign-lang.uni-hamburg.de/dgs-korpus/files/inhalt_pdf/LREC_2012_Colours.pdf

[60] Lopez-Calderon J, Luck SJ. ERPLAB: an open-source toolbox for the analysis of event-related potentials. Front Hum Neurosci. 2014;8: 1–14. 10.3389/fnhum.2014.00001 24782741 PMC3995046

[61] Wittenburg P, Brugman H, Russel A, Klassmann A, Sloetjes H. ELAN: a professional framework for multimodality research. Proceedings of LREC. Genoa, Italy; 2006.

[62] Greenhouse SW, Geisser S. On methods in the analysis of profile data. Psychometrika. 1959;24: 95–112. 10.1007/bf02289823

[63] Friederici AD, Jacobsen T. Processing grammatical gender during language comprehension. J Psycholinguist Res. 1999;28: 467–484. 10.1023/A:1023264209610

[64] Andreassi JL, Okamura H, Stern M. Hemispheric asymmetries in the visual cortical evoked potential as a function of stimulus location. Psychophysiology. 1975;12: 541–546. 10.1111/j.1469-8986.1975.tb00043.x 1181607

[65] Rugg MD, Lines CR, Milner AD. Further investigation of visual evoked potentials elicited by lateralized stimuli: effects of stimulus eccentricity and reference site. Electroencephalogr Clin Neurophysiol Potentials Sect. 1985;62: 81–87. 10.1016/0168-5597(85)90019-x 2578946

[66] Holcomb PJ, Coffey SA, Neville HJ. Visual and auditory sentence processing: A developmental analysis using event-related brain potentials. Dev Neuropsychol. 1992;8: 203–241. 10.1080/87565649209540525

[67] Ardal S, Donald MW, Meuter R, Muldrew S, Luce M. Brain responses to semantic incongruity in bilinguals. Brain Lang. 1990;39: 187–205. 10.1016/0093-934X(90)90011-5 2224493

[68] McLaughlin J, Osterhout L, Kim A. Neural correlates of second-language word learning: Minimal instruction produces rapid change. Nat Neurosci. 2004;7: 703–704. 10.1038/nn1264 15195094

[69] Midgley KJ, Holcomb PJ, Grainger J. Language effects in second language learners and proficient bilinguals investigated with event-related potentials. J Neurolinguistics. 2009;22: 281–300. 10.1016/j.jneuroling.2008.08.001 19430590 PMC2678859

[70] Hosemann J. The processing of German Sign Language sentences. Event-related Potential Studies on Phonological, Morphosyntactic, and Semantic Aspects. [Internet]. Doctoral dissertation, University of Goettingen. 2015. Available: https://ediss.uni-goettingen.de

[71] Kubus O, Villwock A, Morford JP, Rathmann C. Word recognition in deaf readers: Cross-language activation of German Sign Language and German. Appl Psycholinguist. 2015;36: 831–854. 10.1017/S0142716413000520

[72] Thierry G, Wu YJ. Brain potentials reveal unconscious translation during foreign-language comprehension. Proc Natl Acad Sci. 2007;104: 12530–12535. 10.1073/pnas.0609927104 17630288 PMC1941503

[73] van Herten M, Chwilla DJ, Kolk HH. When heuristics clash with parsing routines: ERP evidence for conflict monitoring in sentence perception. J Cogn Neurosci. 2006;18: 1181–1197. 10.1162/jocn.2006.18.7.1181 16839291

[74] Hosemann J, Herrmann A, Sennhenn-Reulen H, Schlesewsky M, Steinbach M. Agreement or no agreement. ERP correlates of verb agreement violation in German Sign Language. Lang Cogn Neurosci. 2018; 1–21. 10.1080/23273798.2018.1465986

[75] Geraci C. Spatial syntax in your hands. In: Iyer J, Kusmer L, editors. Proceedings of the Forty-Fourth Annual Meeting of the North East Linguistic Society. Amherst, MA: GLSA; 2014. pp. 123–134. Available: https://sites.google.com/site/carlogeraci76/home

