# Supplementary material for: Pointing to the right side? An ERP study on anaphora resolution in German Sign Language
Source: PLoS One. 2018 Sep 20;13(9):e0204223. doi: 10.1371/journal.pone.0204223 (PMC6147481; doi:10.1371/journal.pone.0204223)
Supplement: S1 Text — (PDF) [file pone.0204223.s001.pdf]

S1 Text. Notational conventions for sign glosses. By convention, signs are glossed in small caps. In the discussed examples, INDEX is always used as a pronoun to refer back to one of the two DRs. Subscript number '3' refers to the R-locus and subscripts 'a' (right/ipsilateral) and 'b' (left/contralateral) refer to the corresponding region in the horizontal plane. Note that DGS is an SOV language.
