# Supplementary material for: Pointing to the right side? An ERP study on anaphora resolution in German Sign Language
Source: PLoS One. 2018 Sep 20;13(9):e0204223. doi: 10.1371/journal.pone.0204223 (PMC6147481; doi:10.1371/journal.pone.0204223)

S1 Fig. Scene stills of videos for each condition. Lines of different colors indicate the conditions (orange = Condition 1, green = Condition 2, blue = Condition 3, purple = Condition 4). Please follow lines of the same color to get the sequence of video shown for the corresponding condition.

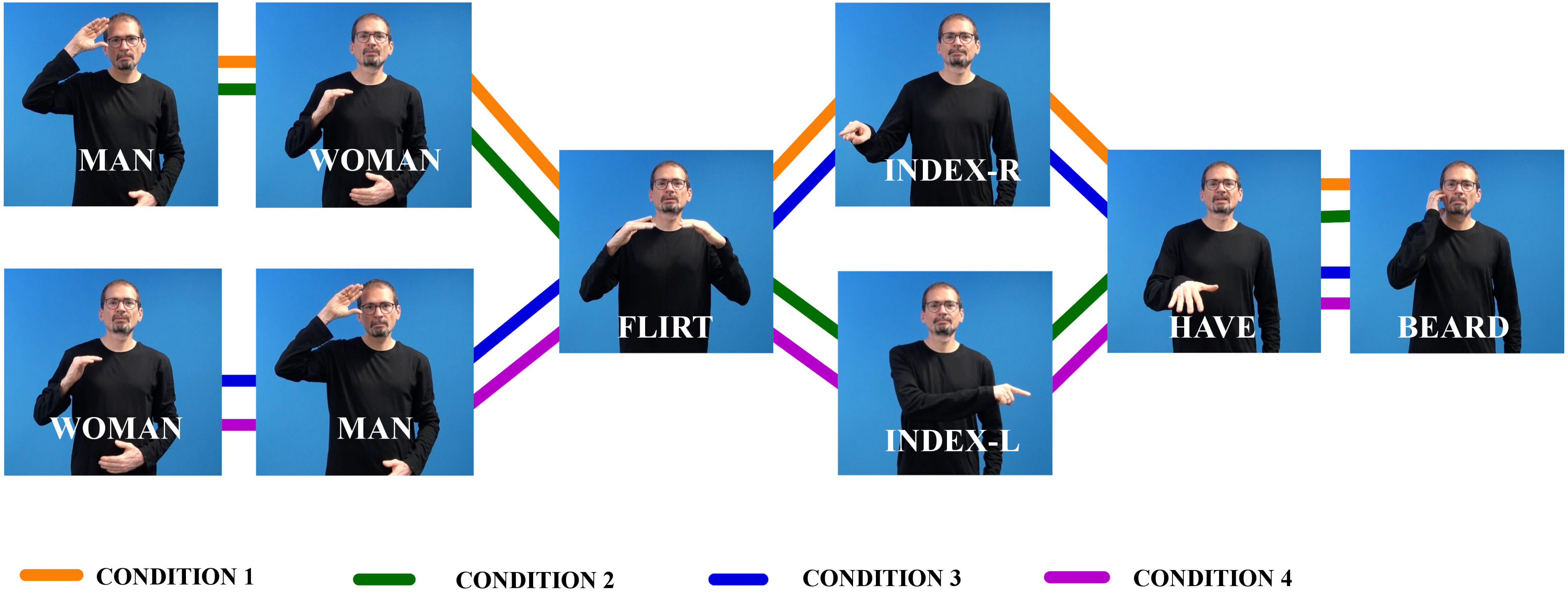

Supplement: S1 Fig — (PDF) [file pone.0204223.s002.pdf]
