# Supplementary material for: Pointing to the right side? An ERP study on anaphora resolution in German Sign Language
Source: PLoS One. 2018 Sep 20;13(9):e0204223. doi: 10.1371/journal.pone.0204223 (PMC6147481; doi:10.1371/journal.pone.0204223)
Supplement: S1 Table — (PDF) [file pone.0204223.s003.pdf]

S1 Table. Full list of stimulus material.

Set	Condition	Direction of the INDEX sign	Congruence of the disambiguating sign	Stimulus material: Glosses of German Sign Language sentences (English translation)
1	1	ipsilateral	congruent	CHILD MOTHER KISS. IX$_{3a}$ ALWAYS CHEEKY.
	2	contralateral	incongruent	CHILD MOTHER KISS. IX$_{3b}$ ALWAYS CHEEKY.
	3	ipsilateral	incongruent	MOTHER CHILD KISS. IX$_{3a}$ ALWAYS CHEEKY.
	4	contralateral	congruent	MOTHER CHILD KISS. IX$_{3b}$ ALWAYS CHEEKY.
2	1	ipsilateral	congruent	WOMAN MAN MEET. IX$_{3a}$ AGAIN PREGNANT.
	2	contralateral	incongruent	WOMAN MAN MEET. IX$_{3b}$ AGAIN PREGNANT.
	3	ipsilateral	incongruent	MAN WOMAN MEET. IX$_{3a}$ AGAIN PREGNANT.
	4	contralateral	congruent	MAN WOMAN MEET. IX$_{3b}$ AGAIN PREGNANT.
3	1	ipsilateral	congruent	COWBOY CHILD SEARCH. IX$_{3a}$ HAVE MOUSTACHE.
	2	contralateral	incongruent	COWBOY CHILD SEARCH. IX$_{3b}$ HAVE MOUSTACHE.
	3	ipsilateral	incongruent	CHILD COWBOY SEARCH. IX$_{3a}$ HAVE MOUSTACHE.
	4	contralateral	congruent	CHILD COWBOY SEARCH. IX$_{3b}$ HAVE MOUSTACHE.
4	1	ipsilateral	congruent	KING WITCH GET-TO-KNOW. IX$_{3a}$ COUNTRY RULE.
	2	contralateral	incongruent	KING WITCH GET-TO-KNOW. IX$_{3b}$ COUNTRY RULE.
	3	ipsilateral	incongruent	WITCH KING GET-TO-KNOW. IX$_{3a}$ COUNTRY RULE.
	4	contralateral	congruent	WITCH KING GET-TO-KNOW. IX$_{3b}$ COUNTRY RULE.
5	1	ipsilateral	congruent	CHILD DOG PLAY. IX$_{3a}$ REGULARLY LAUGH.
	2	contralateral	incongruent	CHILD DOG PLAY. IX$_{3b}$ REGULARLY LAUGH.
	3	ipsilateral	incongruent	DOG CHILD PLAY. IX$_{3a}$ REGULARLY LAUGH.
	4	contralateral	congruent	DOG CHILD PLAY. IX$_{3b}$ REGULARLY LAUGH.
6	1	ipsilateral	congruent	WOMAN BOY CHEEK-KISS. IX$_{3a}$ SOON GIVE-BIRTH.
	2	contralateral	incongruent	WOMAN BOY CHEEK-KISS. IX$_{3b}$ SOON GIVE-BIRTH.
	3	ipsilateral	incongruent	BOY WOMAN CHEEK-KISS. IX$_{3a}$ SOON GIVE-BIRTH.
	4	contralateral	congruent	BOY WOMAN CHEEK-KISS. IX$_{3b}$ SOON GIVE-BIRTH.
7	1	ipsilateral	congruent	MAN CHILD SEARCH. IX$_{3a}$ POSS$_1$ UNCLE.
	2	contralateral	incongruent	MAN CHILD SEARCH. IX$_{3b}$ POSS$_1$ UNCLE.
	3	ipsilateral	incongruent	CHILD MAN SEARCH. IX$_{3a}$ POSS$_1$ UNCLE.
	4	contralateral	congruent	CHILD MAN SEARCH. IX$_{3b}$ POSS$_1$ UNCLE.
8	1	ipsilateral	congruent	BOY GIRL FLIRT. IX$_{3a}$ HAVE TIE.
	2	contralateral	incongruent	BOY GIRL FLIRT. IX$_{3b}$ HAVE TIE.
	3	ipsilateral	incongruent	GIRL BOY FLIRT. IX$_{3a}$ HAVE TIE.
	4	contralateral	congruent	GIRL BOY FLIRT. IX$_{3b}$ HAVE TIE.
9	1	ipsilateral	congruent	GRANDPA BOY GREET. IX$_{3a}$ DAILY PIPE-SMOKE.
	2	contralateral	incongruent	GRANDPA BOY GREET. IX$_{3b}$ DAILY PIPE-SMOKE.
	3	ipsilateral	incongruent	BOY GRANDPA GREET. IX$_{3a}$ DAILY PIPE-SMOKE.
	4	contralateral	congruent	BOY GRANDPA GREET. IX$_{3b}$ DAILY PIPE-SMOKE.
10	1	ipsilateral	congruent	NURSE BABY LIKE. IX$_{3a}$ POSS$_1$ MOTHER.
	2	contralateral	incongruent	NURSE BABY LIKE. IX$_{3b}$ POSS$_1$ MOTHER.
	3	ipsilateral	incongruent	BABY NURSE LIKE. IX$_{3a}$ POSS$_1$ MOTHER.
	4	contralateral	congruent	BABY NURSE LIKE. IX$_{3b}$ POSS$_1$ MOTHER.

11	1	ipsilateral	congruent	FATHER CHILD CHEEK-KISS. IX_{3a} DAILY SHAVE.
	2	contralateral	incongruent	FATHER CHILD CHEEK-KISS. IX_{3b} DAILY SHAVE.
	3	ipsilateral	incongruent	CHILD FATHER CHEEK-KISS. IX_{3a} DAILY SHAVE.
	4	contralateral	congruent	CHILD FATHER CHEEK-KISS. IX_{3b} DAILY SHAVE.
12	1	ipsilateral	congruent	BABY GRANDMA SEE. IX_{3a} ALWAYS SUCK.
	2	contralateral	incongruent	BABY GRANDMA SEE. IX_{3b} ALWAYS SUCK.
	3	ipsilateral	incongruent	GRANDMA BABY SEE. IX_{3a} ALWAYS SUCK.
	4	contralateral	congruent	GRANDMA BABY SEE. IX_{3b} ALWAYS SUCK.
13	1	ipsilateral	congruent	NURSE SOLDIER MARRY. IX_{3a} NOW PREGNANT.
	2	contralateral	incongruent	NURSE SOLDIER MARRY. IX_{3b} NOW PREGNANT.
	3	ipsilateral	incongruent	SOLDIER NURSE MARRY. IX_{3a} NOW PREGNANT.
	4	contralateral	congruent	SOLDIER NURSE MARRY. IX_{3b} NOW PREGNANT.
14	1	ipsilateral	congruent	DOCTOR CHILD SEE. IX_{3a} LONG OPERATE.
	2	contralateral	incongruent	DOCTOR CHILD SEE. IX_{3b} LONG OPERATE.
	3	ipsilateral	incongruent	CHILD DOCTOR SEE. IX_{3a} LONG OPERATE.
	4	contralateral	congruent	CHILD DOCTOR SEE. IX_{3b} LONG OPERATE.
15	1	ipsilateral	congruent	WOMAN BABY LIKE. IX_{3a} EVENINGS READ-ALOUD.
	2	contralateral	incongruent	WOMAN BABY LIKE. IX_{3b} EVENINGS READ-ALOUD.
	3	ipsilateral	incongruent	BABY WOMAN LIKE. IX_{3a} EVENINGS READ-ALOUD.
	4	contralateral	congruent	BABY WOMAN LIKE. IX_{3b} EVENINGS READ-ALOUD.
16	1	ipsilateral	congruent	BOY GIRL KISS. IX_{3a} $POSS_1$ BROTHER.
	2	contralateral	incongruent	BOY GIRL KISS. IX_{3b} $POSS_1$ BROTHER.
	3	ipsilateral	incongruent	GIRL BOY KISS. IX_{3a} $POSS_1$ BROTHER.
	4	contralateral	congruent	GIRL BOY KISS. IX_{3b} $POSS_1$ BROTHER.
17	1	ipsilateral	congruent	WOMAN CHILD GET-TO-KNOW. IX_{3a} RESTAURANT WORK.
	2	contralateral	incongruent	WOMAN CHILD GET-TO-KNOW. IX_{3b} RESTAURANT WORK.
	3	ipsilateral	incongruent	CHILD WOMAN GET-TO-KNOW. IX_{3a} RESTAURANT WORK.
	4	contralateral	congruent	CHILD WOMAN GET-TO-KNOW. IX_{3b} RESTAURANT WORK.
18	1	ipsilateral	congruent	NURSE BOY MEET. IX_{3a} REGULARLY PAINT-FINGERNAILS.
	2	contralateral	incongruent	NURSE BOY MEET. IX_{3b} REGULARLY PAINT-FINGERNAILS.
	3	ipsilateral	incongruent	BOY NURSE MEET. IX_{3a} REGULARLY PAINT-FINGERNAILS.
	4	contralateral	congruent	BOY NURSE MEET. IX_{3b} REGULARLY PAINT-FINGERNAILS.
19	1	ipsilateral	congruent	HEADMASTER PUPIL GREET. IX_{3a} $POSS_1$ TEACHER.
	2	contralateral	incongruent	HEADMASTER PUPIL GREET. IX_{3b} $POSS_1$ TEACHER.
	3	ipsilateral	incongruent	PUPIL HEADMASTER GREET. IX_{3a} $POSS_1$ TEACHER.
	4	contralateral	congruent	PUPIL HEADMASTER GREET. IX_{3b} $POSS_1$ TEACHER.
20	1	ipsilateral	congruent	GRANDPA BOY LIKE. IX_{3a} ALWAYS WALK-WITH-STICK.
	2	contralateral	incongruent	GRANDPA BOY LIKE. IX_{3b} ALWAYS WALK-WITH-STICK.
	3	ipsilateral	incongruent	BOY GRANDPA LIKE. IX_{3a} ALWAYS WALK-WITH-STICK.
	4	contralateral	congruent	BOY GRANDPA LIKE. IX_{3b} ALWAYS WALK-WITH-STICK.
21	1	ipsilateral	congruent	PILOT GIRL GREET. IX_{3a} NOW RETIRED.
	2	contralateral	incongruent	PILOT GIRL GREET. IX_{3b} NOW RETIRED.
	3	ipsilateral	incongruent	GIRL PILOT GREET. IX_{3a} NOW RETIRED.
	4	contralateral	congruent	GIRL PILOT GREET. IX_{3b} NOW RETIRED.
22	1	ipsilateral	congruent	TEACHER PUPIL KNOW. IX_{3a} BACK-THEN STRICT.
	2	contralateral	incongruent	TEACHER PUPIL KNOW. IX_{3b} BACK-THEN STRICT.
	3	ipsilateral	incongruent	PUPIL TEACHER KNOW. IX_{3a} BACK-THEN STRICT.
	4	contralateral	congruent	PUPIL TEACHER KNOW. IX_{3b} BACK-THEN STRICT.

23	1	ipsilateral	congruent	PRIEST GRANDMA MEET. IX$_{3a}$ HAVE BEARD.
	2	contralateral	incongruent	PRIEST GRANDMA MEET. IX$_{3b}$ HAVE BEARD.
	3	ipsilateral	incongruent	GRANDMA PRIEST MEET. IX$_{3a}$ HAVE BEARD.
	4	contralateral	congruent	GRANDMA PRIEST MEET. IX$_{3b}$ HAVE BEARD.
24	1	ipsilateral	congruent	COWBOY DOG SEE. IX$_{3a}$ HAVE GUN.
	2	contralateral	incongruent	COWBOY DOG SEE. IX$_{3b}$ HAVE GUN.
	3	ipsilateral	incongruent	DOG COWBOY SEE. IX$_{3a}$ HAVE GUN.
	4	contralateral	congruent	DOG COWBOY SEE. IX$_{3b}$ HAVE GUN.
25	1	ipsilateral	congruent	GIRL BOY CHEEK-KISS. IX$_{3a}$ ALWAYS PUT-LIPSTICK-ON.
	2	contralateral	incongruent	GIRL BOY CHEEK-KISS. IX$_{3b}$ ALWAYS PUT-LIPSTICK-ON.
	3	ipsilateral	incongruent	BOY GIRL CHEEK-KISS. IX$_{3a}$ ALWAYS PUT-LIPSTICK-ON.
	4	contralateral	congruent	BOY GIRL CHEEK-KISS. IX$_{3b}$ ALWAYS PUT-LIPSTICK-ON.
26	1	ipsilateral	congruent	WOMAN MAN MARRY. IX$_{3a}$ POSS$_1$ SISTER.
	2	contralateral	incongruent	WOMAN MAN MARRY. IX$_{3b}$ POSS$_1$ SISTER.
	3	ipsilateral	incongruent	MAN WOMAN MARRY. IX$_{3a}$ POSS$_1$ SISTER.
	4	contralateral	congruent	MAN WOMAN MARRY. IX$_{3b}$ POSS$_1$ SISTER.
27	1	ipsilateral	congruent	NURSE MAN FLIRT. IX$_{3a}$ ALWAYS HIGH-HEELS-WEAR.
	2	contralateral	incongruent	NURSE MAN FLIRT. IX$_{3b}$ ALWAYS HIGH-HEELS-WEAR.
	3	ipsilateral	incongruent	MAN NURSE FLIRT. IX$_{3a}$ ALWAYS HIGH-HEELS-WEAR.
	4	contralateral	congruent	MAN NURSE FLIRT. IX$_{3b}$ ALWAYS HIGH-HEELS-WEAR.
28	1	ipsilateral	congruent	SOLDIER CHILD MEET. IX$_{3a}$ REGULARLY CAR-DRIVE.
	2	contralateral	incongruent	SOLDIER CHILD MEET. IX$_{3b}$ REGULARLY CAR-DRIVE.
	3	ipsilateral	incongruent	CHILD SOLDIER MEET. IX$_{3a}$ REGULARLY CAR-DRIVE.
	4	contralateral	congruent	CHILD SOLDIER MEET. IX$_{3b}$ REGULARLY CAR-DRIVE.
29	1	ipsilateral	congruent	BOY CAT SEE. IX$_{3a}$ NOW SIGN.
	2	contralateral	incongruent	BOY CAT SEE. IX$_{3b}$ NOW SIGN.
	3	ipsilateral	incongruent	CAT BOY SEE. IX$_{3a}$ NOW SIGN.
	4	contralateral	congruent	CAT BOY SEE. IX$_{3b}$ NOW SIGN.
30	1	ipsilateral	congruent	MAN WOMAN FLIRT. IX$_{3a}$ HAVE BEARD.
	2	contralateral	incongruent	MAN WOMAN FLIRT. IX$_{3b}$ HAVE BEARD.
	3	ipsilateral	incongruent	WOMAN MAN FLIRT. IX$_{3a}$ HAVE BEARD.
	4	contralateral	congruent	WOMAN MAN FLIRT. IX$_{3b}$ HAVE BEARD.
31	1	ipsilateral	congruent	GIRL CAT PLAY. IX$_{3a}$ ALWAYS SPEAK.
	2	contralateral	incongruent	GIRL CAT PLAY. IX$_{3b}$ ALWAYS SPEAK.
	3	ipsilateral	incongruent	CAT GIRL PLAY. IX$_{3a}$ ALWAYS SPEAK.
	4	contralateral	congruent	CAT GIRL PLAY. IX$_{3b}$ ALWAYS SPEAK.
32	1	ipsilateral	congruent	MAN WOMAN KISS. IX$_{3a}$ BACK-THEN OFFICER.
	2	contralateral	incongruent	MAN WOMAN KISS. IX$_{3b}$ BACK-THEN OFFICER.
	3	ipsilateral	incongruent	WOMAN MAN KISS. IX$_{3a}$ BACK-THEN OFFICER.
	4	contralateral	congruent	WOMAN MAN KISS. IX$_{3b}$ BACK-THEN OFFICER.
33	1	ipsilateral	congruent	PROFESSOR GRANDMA GET-TO-KNOW. IX$_{3a}$ POSS$_1$ LECTURER.
	2	contralateral	incongruent	PROFESSOR GRANDMA GET-TO-KNOW. IX$_{3b}$ POSS$_1$ LECTURER.
	3	ipsilateral	incongruent	GRANDMA PROFESSOR GET-TO-KNOW. IX$_{3a}$ POSS$_1$ LECTURER.
	4	contralateral	congruent	GRANDMA PROFESSOR GET-TO-KNOW. IX$_{3b}$ POSS$_1$ LECTURER.
34	1	ipsilateral	congruent	BOY CAT PLAY. IX$_{3a}$ OFTEN READ.
	2	contralateral	incongruent	BOY CAT PLAY. IX$_{3b}$ OFTEN READ.
	3	ipsilateral	incongruent	CAT BOY PLAY. IX$_{3a}$ OFTEN READ.
	4	contralateral	congruent	CAT BOY PLAY. IX$_{3b}$ OFTEN READ.

35	1	ipsilateral	congruent	WOMAN CHILD CHEEK-KISS. IX_{3a} SOON GRANDMAN.
	2	contralateral	incongruent	WOMAN CHILD CHEEK-KISS. IX_{3b} SOON GRANDMAN.
	3	ipsilateral	incongruent	CHILD WOMAN CHEEK-KISS. IX_{3a} SOON GRANDMAN.
	4	contralateral	congruent	CHILD WOMAN CHEEK-KISS. IX_{3b} SOON GRANDMAN.
36	1	ipsilateral	congruent	GIRL BABY LIKE. IX_{3a} REGULARLY SKIP-ROPE.
	2	contralateral	incongruent	GIRL BABY LIKE. IX_{3b} REGULARLY SKIP-ROPE.
	3	ipsilateral	incongruent	BABY GIRL LIKE. IX_{3a} REGULARLY SKIP-ROPE.
	4	contralateral	congruent	BABY GIRL LIKE. IX_{3b} REGULARLY SKIP-ROPE.
37	1	ipsilateral	congruent	MAN CHILD GREET. IX_{3a} SOON DIVORCED.
	2	contralateral	incongruent	MAN CHILD GREET. IX_{3b} SOON DIVORCED.
	3	ipsilateral	incongruent	CHILD MAN GREET. IX_{3a} SOON DIVORCED.
	4	contralateral	congruent	CHILD MAN GREET. IX_{3b} SOON DIVORCED.
38	1	ipsilateral	congruent	BABY GRANDPA KISS. IX_{3a} NOW CRAWL.
	2	contralateral	incongruent	BABY GRANDPA KISS. IX_{3b} NOW CRAWL.
	3	ipsilateral	incongruent	GRANDPA BABY KISS. IX_{3a} NOW CRAWL.
	4	contralateral	congruent	GRANDPA BABY KISS. IX_{3b} NOW CRAWL.
39	1	ipsilateral	congruent	NUN GIRL GET-TO-KNOW. IX_{3a} $POSS_1$ AUNT.
	2	contralateral	incongruent	NUN GIRL GET-TO-KNOW. IX_{3b} $POSS_1$ AUNT.
	3	ipsilateral	incongruent	GIRL NUN GET-TO-KNOW. IX_{3a} $POSS_1$ AUNT.
	4	contralateral	congruent	GIRL NUN GET-TO-KNOW. IX_{3b} $POSS_1$ AUNT.
40	1	ipsilateral	congruent	WOMAN PILOT FLIRT. IX_{3a} HAVE SKIRT.
	2	contralateral	incongruent	WOMAN PILOT FLIRT. IX_{3b} HAVE SKIRT.
	3	ipsilateral	incongruent	PILOT WOMAN FLIRT. IX_{3a} HAVE SKIRT.
	4	contralateral	congruent	PILOT WOMAN FLIRT. IX_{3b} HAVE SKIRT.
